# Changes in susceptibility to life-threatening infections after treatment for complicated severe malnutrition in Kenya

**DOI:** 10.1093/ajcn/nqy007

**Published:** 2018-04-09

**Authors:** Moses M Ngari, Laura Mwalekwa, Molline Timbwa, Fauzat Hamid, Rehema Ali, Per Ole Iversen, Greg W Fegan, James A Berkley

**Affiliations:** 1 KEMRI/Wellcome Trust Research Programme, Kilifi, Kenya; 2 The Childhood Acute Illness and Nutrition (CHAIN) Network, Nairobi, Kenya; 3 Department of Nutrition, IBM, University of Oslo, Norway; 4 Department of Hematology, Oslo University Hospital, Oslo, Norway; 5 Swansea Trials Unit, Swansea University Medical School, Swansea, United Kingdom; 6 Center for Tropical Medicine and Global Health, University of Oxford, Oxford, United Kingdom

**Keywords:** malnutrition, SAM, children, recovery, life-threatening infections, mortality

## Abstract

**Background:**

Goals of treating childhood severe acute malnutrition (SAM), in addition to anthropometric recovery and preventing short-term mortality, include reducing the risks of subsequent serious infections. How quickly and how much the risk of serious illness changes during rehabilitation are unknown but could inform improving the design and scope of interventions.

**Objective:**

The aim of this study was to investigate changes in the risk of life-threatening events (LTEs) in relation to anthropometric recovery from SAM.

**Design:**

This was a secondary analysis of a clinical trial including 1778 HIV-uninfected Kenyan children aged 2–59 mo with complicated SAM, enrolled after the inpatient stabilization phase of treatment, and followed for 12 mo. The main outcome was LTEs, defined as infections requiring rehospitalization or causing death. We examined anthropometric variables measured at months 1, 3, and 6 after enrollment in relation to LTEs occurring during the 6 mo after each of these time points.

**Results:**

Over 12 mo, there were 823 LTEs (257 fatal), predominantly severe pneumonia and diarrhea. At months 1, 3, and 6, 557 (34%), 764 (49%), and 842 (56%) children had a weight-for-height or -length *z* score (WHZ) ≥−2, respectively, which, compared with a WHZ <−3, was associated with lower risks of subsequent LTEs [adjusted HRs (95% CIs): 0.50 (0.40, 0.64), 0.30 (0.23, 0.39), and 0.23 (0.16, 0.32), respectively]. However, children with a WHZ ≥−2 at 1, 3, and 6 mo still had 39 (95% CI: 32, 47), 26 (95% CI: 22, 32), and 15 (95% CI: 12, 20) LTEs/100 child-years of observation during the following 6 mo. WHZ at study enrollment predicted subsequent WHZ but not the risk of LTEs. Changes in height-for-age *z* score did not predict LTEs.

**Conclusions:**

Anthropometric response was associated with a rapid and substantial reduction in risk of LTEs. However, reduction in susceptibility lagged behind anthropometric improvement. Disease events, together with anthropometric assessment, may provide a clearer picture of the effectiveness of interventions. Robust protocols for detecting and treating poor anthropometric recovery and addressing broader vulnerabilities that complicated SAM indicates may save lives. This trial was registered at www.clinicaltrials.gov as NCT00934492.

## INTRODUCTION

Severe acute malnutrition (SAM) affects ≥16 million children aged <5 y, ∼1 million of whom die annually, predominantly from common infections (1–3). Pooled global data suggest that the risk of death from all causes, diarrhea, or pneumonia among children in the community with SAM is ∼9-, 13-, and 8-fold higher than among well-nourished children, respectively (1, 4).

The treatment of SAM involves treating infections, therapeutic feeding, correcting metabolic and electrolyte imbalances, and psychosocial support to prevent short-term mortality and achieve catch-up growth (5). SAM with infectious complications is usually treated in the hospital, although children with uncomplicated SAM are treated as outpatients.

Important longer-term goals of the treatment of SAM, in addition to anthropometric recovery, are reducing the susceptibility to life-threatening infections, restoring a healthy body composition, and improving neurocognitive status (5). However, severe illness and mortality are well recognized as occurring after treatment of SAM or moderate acute malnutrition (6–9). It is conceivable that anthropometric recovery and reduced susceptibility to infection may exhibit different temporal patterns, and thus simple anthropometric measurements might not directly reflect immediate risk. Although several studies have examined pediatric postdischarge mortality in developing countries, to our knowledge no study has examined the risk and timing of life-threatening illness during and after recovery from complicated SAM treated in the hospital in low-resource settings (10). Such knowledge could provide evidence to inform recommendations for follow-up to reduce the overall mortality associated with SAM.

We aimed in this study to determine changes in the risk of life-threatening events (LTEs) in relation to anthropometric status during recovery from SAM. We performed a secondary analysis of data from a randomized controlled trial amongst 1778 HIV-uninfected children admitted to the hospital with complicated SAM, who were treated and closely followed up for new infectious disease episodes for 1 y.

## METHODS

### Study design

We performed a secondary observational cohort analysis of data from a randomized controlled trial investigating the efficacy of daily cotrimoxazole prophylaxis in reducing long-term mortality among HIV-uninfected children with SAM admitted to 4 hospitals in Kenya (7). In the parent trial, participants were randomly assigned to receive daily cotrimoxazole prophylaxis or placebo for the first 6 mo and followed up for 12 mo, as described elsewhere (7). There was no evidence that daily cotrimoxazole prophylaxis reduced mortality or improved growth among this population in the primary trial (7).

### Study setting

Children were recruited in the pediatric wards of 3 secondary hospitals—Kilifi County Hospital (rural), Malindi subcounty hospital (rural), and the Mbagathi subcounty hospital in Nairobi (urban)—and 1 tertiary-level hospital, Coast General Hospital in Mombasa (urban). Inpatient care for SAM and other conditions and subsequent outpatient care was provided according to WHO and Kenyan national guidelines (5).

### Study population

The parent trial participants were children aged 2–59 mo who had a negative HIV rapid antibody test and who were admitted to the hospital with SAM, defined as midupper arm circumference (MUAC) <11.0 cm (children aged 2–6 mo) or MUAC <11.5 cm (children aged 6–59 mo) or the presence of kwashiorkor at any age (7). The more traditional method of assessing childhood malnutrition, weight-for-height or -length *z* score (WHZ), was not used for trial entry because MUAC predicts mortality better than WHZ (11). HIV-infected children were excluded from the parent trial because daily cotrimoxazole prophylaxis is already standard of care for this group. Participants were enrolled once they had completed the stabilization phase of inpatient care, as defined by the WHO, and discharged following WHO guidelines (5). At hospital discharge, nutritional counseling was given to caregivers, along with ready-to-use therapeutic food, and families were referred to community-based management of acute malnutrition centers located either at the hospital or in community facilities, if these were more accessible, to continue with outpatient therapeutic feeding.

### Variables

The primary outcomes of interest were LTEs, defined as infections occurring after discharge from the index hospital admission requiring rehospitalization or causing death. LTEs were diagnosed and recorded by trained study clinicians according to the WHO 2005 clinical definitions (5).

To determine the associations between anthropometric attainment during follow-up and subsequent LTEs, we examined the exposures of nutritional status defined by WHZ, absolute MUAC, and height- or length-for-age *z* score (HAZ) after 1, 3, and 6 mo, and the demographic, clinical, and anthropometric characteristics at study enrollment.

To maintain uniformity in follow-up time for analysis at each of these time points and to maximize the duration analyzed within the overall 12-mo follow-up period, we identified the LTEs that occurred during 6 mo after each time point. Thus, the analysis of anthropometric status at month 1 was in relation to LTEs occurring from months 1 to 7. Similarly, for anthropometric status at months 3 and 6, LTEs were analyzed from months 3 to 9 and from months 6 to 12 (the end of trial follow-up), respectively.

Because children were enrolled based on MUAC, they had a range of WHZ and HAZ values at study enrollment. We performed the primary analysis using WHZ because its calculation is age-independent and it is the standard measure of anthropometric recovery used by the WHO (12, 13). We also examined absolute MUAC because this was the enrollment criterion (in addition to kwashiorkor), and MUAC-only programs are increasingly utilized (7). Kwashiorkor was present in 17% of children and included in the study enrollment analysis, but was not considered a separate factor in the analyses performed at 1, 3, or 6 mo because it was rarely present at follow-up (1.1%, 0.2%, and 0.2% at 1, 3, and 6 mo, respectively), and WHZ was treated as “missing” at these time points if kwashiorkor was present. We also examined LTEs after a missing WHZ value, because this may be a source of bias where nonattendance at follow-up is associated with illness, lack of access, or socioeconomic disadvantage.

### Data sources and measurement

At enrollment, child and caregiver demographic characteristics, immunization status, clinical examination, admission diagnoses, chronic conditions, and anthropometric measurements (weight, height or length, MUAC) were collected. Children’s weights were measured with the use of an electronic scale (Seca 825), length or height with the use of an infantometer (Seca 416) or stadiometer (Seca 215), and MUAC with the use of insertion tape (TALC) (7). We calculated anthropometric *z* scores at study enrollment and at 1, 3, and 6 mo with the use of the 2006 WHO growth references. Severe anemia was defined as a hemoglobin concentration <5 g/dL.

The children were followed up at the study clinics monthly for the first 6 mo, and then bimonthly through month 12. We collected history of any readmission to the 4 study sites hospitals and any nonstudy hospitals at each visit. In addition, free walk-in clinics were provided at the study hospitals for unscheduled visits where minor illnesses were treated on an outpatient basis or children were referred for hospital admission in case of severe illness. Details of readmissions to hospital or deaths were recorded directly at the study hospitals, from discharge letters, from the medical records for admissions to nonstudy hospitals, or from death certificates. When a child did not attend the study clinic at the scheduled follow-up, they were traced in the community, vital status and history of hospitalizations were sought, and MUAC was measured. In the event of death outside a study hospital, verbal autopsies were conducted after a culturally appropriate interval. Causes of death were assigned by 2 independent pediatricians after review of each child's available information (7).

### Study size

Given the fixed nature of study population from the parent trial, no formal sample size determination for this secondary analysis was conducted. We used the sample size calculated in the parent trial, which had 90% power to detect a one-third reduction in mortality, accounting for loss to follow-up, assuming 15% of children in the control arm would die. A total of 1778 participants were enrolled into the trial. Overall, there were 823 LTEs among 612 participants.

### Study approvals

The trial and secondary analyses were approved by the Kenya National Ethical Review Committee (SSC 1562) and the Oxford Tropical Research Ethics Committee (OXTREC reference 18-09). The trial was registered at clinicaltrials.gov (NCT00934492).

### Statistical methods

Statistical analysis was conducted with the use of Stata version 13.1 (StataCorp). We categorized nutritional status after 1, 3, and 6 months as not wasted (WHZ ≥−2), moderately wasted (WHZ: −3 to −2), and severely wasted (WHZ <−3; reference category). We also examined absolute MUACs of ≥12.5, 11.5–12.4, and <11.5 cm and HAZs ≥−2, −3 to −2.01, and <−3. Where categorical WHZ, MUAC, and HAZ were used in the analysis, an extra group was assigned to denote the missing anthropometric data. Imputation was not performed because children with missing data (e.g., a missed follow-up visit) were anticipated to have a poorer outcome.

The intervention tested in the parent trial was unsuccessful in influencing either mortality or nutritional recovery (7). However, to control for any nonsignificant effects, while including all available children, all of the analyses were adjusted for study group allocation.

We performed a multiple events survival analysis, allowing participants to contribute >1 LTE during the entire 12-mo follow-up, then during the 6-mo periods after the follow-up visits at months 1, 3, and 6. Data were censored at 182 d after each of these visits or at death or loss to follow-up. Incidence rates of LTEs were computed as the number of episodes per child-year of observation (CYO). Observation time was split by using Lexis expansion, and changes in the monthly incidence rate of LTEs during follow-up were tested with the use of an extension of Wilcoxon’s rank-sum test for trend across ordered groups (14).

To examine the effect of nutritional recovery after 1, 3, and 6 mo on the subsequent risk of LTEs during the next 6 mo, we constructed Kaplan-Meier survival plots and used Cox proportional regression to compute HRs, adjusting for study enrollment age, sex, recruitment site, and randomization arm. We computed the HRs for all LTEs, fatal episodes, severe pneumonia, severe diarrhea, and “other” LTEs. To test time lag in recovery, we tested the hypothesis that the HR-associated continuous WHZs were not heterogeneous at months 1, 3, and 6 with the use of meta-analysis with random effects.

We used multinomial logistic regression to analyze the putative study enrollment risk factors for nutritional status (not wasted, moderately wasted, and severely wasted) at months 1, 3, and 6 (15, 16). All of the independent variables hypothesized a priori to be potentially important (age, sex, nutritional status, size at birth, primary caregiver other than the child's mother, economic indicators, and clinical presentation at index admission), as well as enrollment site (a stratification in the parent trial) and study group allocation, were examined in the univariate multinomial logistic regression analysis and retained in the multivariable regression models. We tested for linear trend across the 3 ordered nutritional groups (WHZ: ≥−2, −3 to −2, and <−3) using linear regression with stepwise elimination of independent variables at *P* values ≥0.1, and reported variables with a final *P* value <0.05.

## RESULTS

### Study participants

**FIGURE 1:**
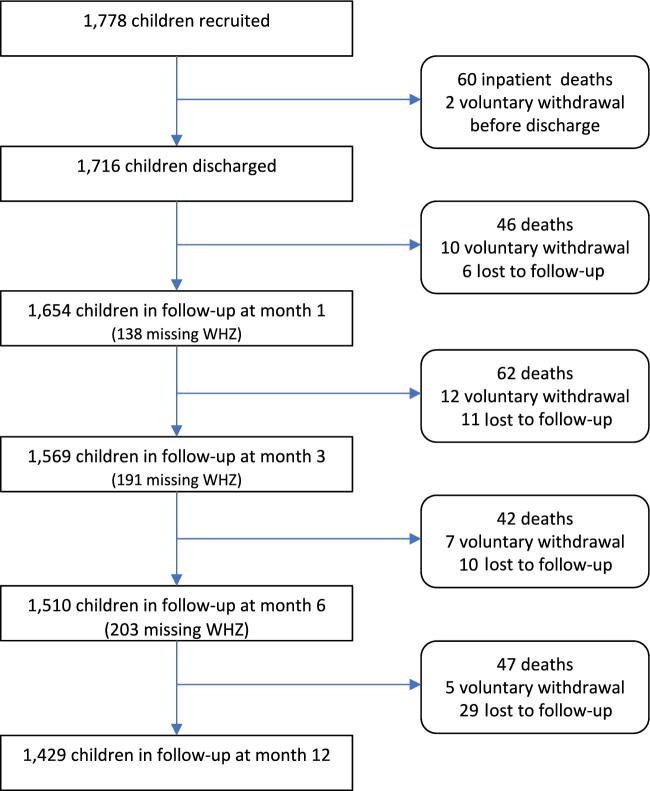
Study flow chart showing numbers of included participants during follow-up at the analysis time points. WHZ, weight-for-height or -length *z* score.

A total of 1778 participants were recruited to the parent trial: 300 (17%) had edema, 908 (51%) had a WHZ <−3, 373 (21%) had a WHZ of −3 to −2, and 197 (11%) had a WHZ ≥−2. A total of 1716 children were discharged and followed up in the community (**Figure 1**). At months 1, 3, and 6, 1654, 1569, and 1510 children were in follow-up, respectively (Figure 1). By the end of follow-up (month 12), 1429 (80%) were in follow-up, 257 (14%) had died, and 92 (5.1%) were either lost to follow-up or voluntarily withdrew from the study (Figure 1). There was no difference in enrollment characteristics between children who completed the study as planned and those lost to follow-up (all *P* > 0.05). Study enrollment characteristics stratified by WHZ category at 3 mo are shown in **Table 1**.

**TABLE 1:** Characteristics of the study participants at study enrollment by nutritional status at month 3^1^

	Nutritional status at 3 mo of follow-up: WHZ			
Characteristic	≥−2 (*n* = 764)	−3 to −2 (*n* = 306)	<−3 (*n* = 308)	*P* ^2^
Female sex, *n* (%)	394 (52)	142 (46)	128 (42)	
Age,^3^ mo	12.0 (7.5, 18.3)	11.2 (7.8, 16.2)	9.9 (6.9, 14.3)	0.03
Nutritional edema, *n* (%)	187 (25)	32 (10)	18 (5.9)	
Midupper arm circumference, cm	10.7 ± 1.1^4^	10.5 ± 0.9	10.3 ± 0.9	
Weight-for-length *z* score	−2.9 ± 1.2	−3.6 ± 1.0	−4.1 ± 1.1	<0.001
Length-for-age *z* score	−2.9 ± 1.6	−2.7 ± 1.5	−2.7 ± 1.7	
Hemoglobin, g/dL	9.7 ± 2.2	9.9 ± 2.5	10.1 ± 2.2	
Clinical signs of rickets, *n* (%)	67 (8.8)	55 (18)	61 (20)	0.003
Known tuberculosis at enrollment, *n* (%)	33 (4.3)	14 (4.6)	6 (2.0)	
Index admission for severe pneumonia *n* (%)	234 (31)	117 (38)	143 (47)	0.03
Index admission for diarrhea, *n* (%)	448 (59)	172 (56)	166 (54)	<0.001
Received conjugate pneumococcal vaccine,^5^*n* (%)	435 (57)	181 (59)	181 (59)	
Born premature/underweight,^6^*n* (%)	165 (22)	65 (21)	67 (22)	
Randomly assigned to cotrimoxazole prophylaxis, *n* (%)	396 (52)	153 (50)	147 (48)	
Urban sites, *n* (%)	536 (70)	237 (77)	268 (88)	
Mother as primary caretaker, *n* (%)	699 (92)	292 (95)	292 (96)	0.02
Primary caretaker had at least primary education,^7^*n* (%)	445 (58)	183 (59)	179 (59)	
Primary caretaker aged <25 y,^7^*n* (%)	221 (29)	68 (22)	71 (23)	
Household with ≥5 persons, *n* (%)	459 (60)	181 (59)	158 (52)	
Using solid fuel indoors, *n* (%)	393 (55)	121 (43)	121 (44)	
House wall material of mud/iron sheet, *n* (%)	397 (52)	144 (47)	104 (34)	
Water source, nontap, *n* (%)	213 (28)	72 (23)	79 (26)	
Type of toilet: community shared/none, *n* (%)	693 (91)	265 (86)	275 (90)	

1 Weight-for-length *z* score excludes children with kwashiorkor. WHZ, weight-for-height or -length *z* score.

2 Linear trend *P* values derived by using stepwise linear regression analysis; variables with *P* ≥ 0.1 were eliminated and those with a final *P* < 0.05 are reported.

3 Values are medians (IQRs).

4 Mean ± SD (all such values).

5 ≥1 dose of conjugate pneumococcal vaccine at enrollment.

6 Gestational age of <37 wk or birth weight <2500 g.

7 Data collected from April 2011.

### Anthropometric recovery

At months 1, 3, 6, and 12, WHZs <−3 were present in 551 (34%), 308 (20%), 204 (13%), and 124 (8.6%) children, respectively. At these time points, 557 (34%), 764 (49%), 842 (56%), and 930 (65%) of children had WHZs ≥−2 (**Supplemental Table 1**). The mean ± SD WHZ, MUAC, and HAZ at the final follow-up at month 12 were −1.16 ± 1.4, 13.5 ± 1.4 cm, and −2.88 ± 1.4, respectively.

Children who had a WHZ ≥−2 at 1 mo had a 90% probability of sustaining this at 3 and 6 mo. However, for children with a WHZ <−3 at 1 mo, only 24% and 37% had a WHZ ≥−2 by 3 and 6 mo, respectively.

### All LTEs during 12 mo of follow-up

After enrollment, there were 823 LTEs (257 deaths and 566 nonfatal events) among 612 of 1778 (34%) participants during 1556 CYOs during the 12-mo follow-up [incidence rate: 0.54 (95% CI: 0.51, 0.58)/CYO] (**Supplemental Table 2**). Of the 823 LTEs, 282 (34%) occurred either in a nonstudy hospital or in the community.

**FIGURE 2:**
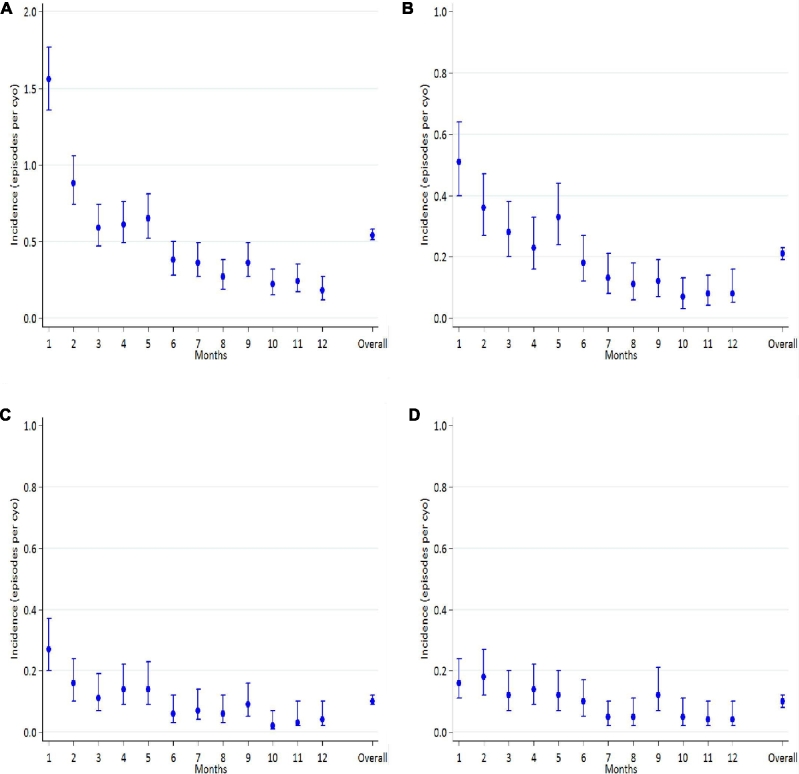
Monthly incidence rates (95% CIs) of any life-threatening event (A), severe pneumonia (B), diarrhea (C), and other life-threatening episodes (D). cyo, child-year of observation.

There were 413, 207, and 274 episodes of severe pneumonia, diarrhea, and other LTEs [incidence rates (95% CIs): 0.21 (0.19, 0.23), 0.10 (0.09, 0.12), and 0.10 (0.08, 0.12) episodes per CYO, respectively]. The 274 episodes of other LTEs were as follows: 53 tuberculosis, 42 urinary tract infections, 21 malaria, 18 severe anemia, 27 sepsis, 15 meningitis, 4 bacteremia, 52 unknown severe febrile illness, and 42 causes of death unknown. The incidence of all LTEs declined during follow-up (all *P*-trend_ _< 0.005; **Figure 2**, Supplemental Table 2).

### Association between nutritional status during follow-up and risk of LTEs

**FIGURE 3:**
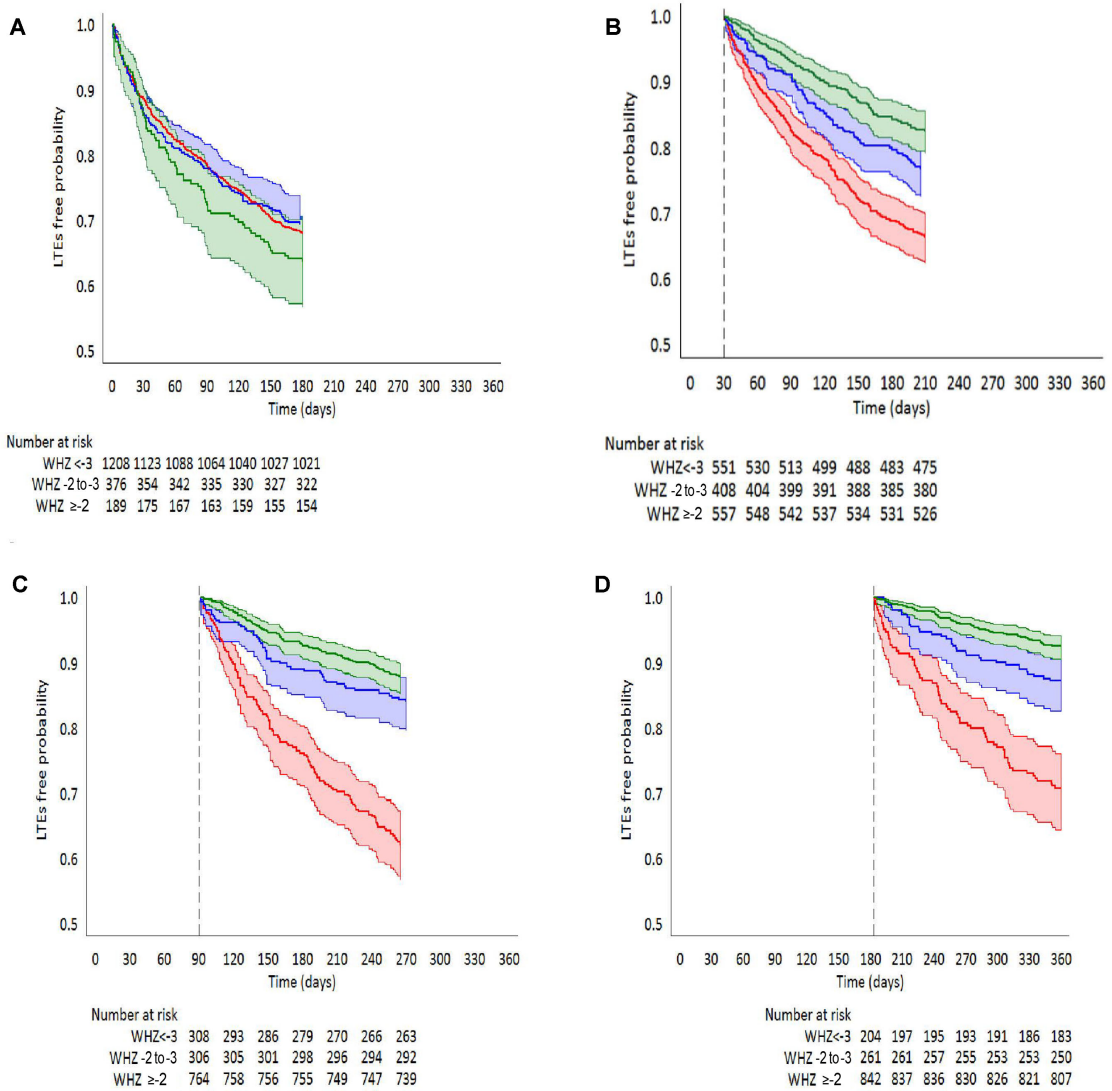
Kaplan-Meier graphs of the probabilities of remaining free of LTEs in the subsequent 6 mo by WHZ (colored areas represent 95% CIs) after study enrollment (A), month 1 (B), month 3 (C), and month 6 (D). WHZ: <−3 (red); −2 to −3 (blue), and ≥−2 (green). LTE, life-threatening event; WHZ, weight-for-height or -length *z* score.

A total of 602 of 823 (73%) of all LTEs occurred after month 1 (**Supplemental Table 3**). The adjusted HRs (aHRs) for LTEs during the 6 mo after the follow-up visits at 1, 3, and 6 mo indicated a reduction in risk by 34%, 62%, and 73%, respectively, for children with WHZs of −3 to −2 and 50%, 70%, and 77%, respectively, for children with WHZs ≥−2, compared with those with WHZs <−3 at each visit (**Table 2**, **Figure 3**).

**TABLE 2:** HRs for LTEs occurring in the subsequent 6 mo by WHZ and absolute MUAC by type of LTE at months 1, 3, and 6 of follow-up^1^

	WHZ (95% CI)				MUAC (95% CI)			
	≥−2	−2 to −3	Missing	<−3	≥12.5 cm	12.5–11.5 cm	Missing	<11.5 cm
At month 1								
All LTEs	0.50 (0.40, 0.64)	0.66 (0.52, 0.84)	0.67 (0.45, 0.99)	1.0	0.37 (0.25, 0.55)	0.59 (0.47, 0.75)	0.88 (0.58, 1.33)	1.0
Death	0.39 (0.24, 0.65)	0.39 (0.22, 0.68)	1.47 (0.86, 2.50)	1.0	0.18 (0.05, 0.59)	0.44 (0.26, 0.75)	2.15 (1.23, 3.77)	1.0
Severe pneumonia	0.51 (0.37, 0.71)	0.63 (0.45, 0.88)	0.37 (019, 0.73)	1.0	0.30 (0.16, 0.59)	0.66 (0.49, 0.91)	0.47 (0.22, 1.01)	1.0
Severe diarrhea	0.33 (0.19, 0.58)	0.78 (0.49, 1.22)	0.43 (0.17, 1.08)	1.0	0.62 (0.26, 1.50)	0.91 (0.58, 1.43)	0.48 (0.15, 1.53)	1.0
Others^2^	0.36 (0.24, 0.56)	0.44 (0.28, 0.69)	0.95 (0.55, 1.64)	1.0	0.29 (0.14, 0.59)	0.48 (0.31, 0.73)	1.50 (0.85, 2.64)	1.0
At month 3								
All LTEs	0.30 (0.23, 0.39)	0.38 (0.28, 0.53)	0.45 (0.31, 0.65)	1.0	0.30 (0.22, 0.40)	0.40 (0.30, 0.53)	0.55 (0.37, 0.82)	1.0
Death	0.16 (0.09, 0.31)	0.32 (0.16, 0.64)	0.70 (0.37, 1.33)	1.0	0.21 (0.10, 0.43)	0.35 (0.19, 0.65)	1.06 (0.56, 2.03)	1.0
Severe pneumonia	0.30 (0.20, 0.43)	0.38 (0.24, 0.60)	0.31 (0.17,0.57)	1.0	0.29 (0.19, 0.44)	0.46 (0.31, 0.67)	0.35 (0.18, 0.67)	1.0
Severe diarrhea	0.29 (0.17, 0.49)	0.53 (0.30, 0.94)	0.09 (0.02, 0.39)	1.0	0.28 (0.15, 0.51)	0.31 (0.18, 0.55)	0.12 (0.03, 0.49)	1.0
Others^2^	0.32 (0.20, 0.51)	0.33 (0.18, 0.60)	0.54 (0.29, 1.01)	1.0	0.25 (0.14, 0.44)	0.48 (0.30, 0.78)	0.81 (0.44, 1.49)	1.0
At month 6								
All LTEs	0.23 (0.16, 0.32)	0.41 (0.27, 0.62)	0.46 (0.27, 0.62)	1.0	0.30 (0.21, 0.44)	0.45 (0.31, 0.67)	0.46 (0.28, 0.75)	1.0
Death	0.11 (0.05, 0.26)	0.44 (0.20, 0.97)	0.69 (0.32, 1.49)	1.0	0.17 (0.07, 0.40)	0.51 (0.24, 1.10)	0.87 (0.38, 1.99)	1.0
Severe pneumonia	0.18 (0.10, 0.30)	0.33 (0.18, 0.61)	0.24 (0.11, 0.52)	1.0	0.26 (0.15, 0.44)	0.32 (0.17, 0.59)	0.28 (0.12, 0.64)	1.0
Severe diarrhea	0.24 (0.12, 0.48)	0.41 (0.18, 0.90)	0.25 (0.08, 0.74)	1.0	0.33 (0.16, 0.69)	0.54 (0.25, 1.18)	0.35 (0.11, 1.07)	1.0
Others^2^	0.15 (0.08, 0.28)	0.30 (0.14, 0.62)	0.58 (0.30, 1.11)	1.0	0.21 (0.11, 0.41)	0.56 (0.29, 1.06)	0.62 (0.29, 1.33)	1.0

1 Values are adjusted HRs (95% CIs). WHZ and MUAC values were adjusted for age, sex, recruitment site, and randomization group. LTE, life-threatening event; MUAC, midupper arm circumference; WHZ, weight-for-height or -length *z* score.

2 “Others” included 4 cases of bacteremia, 15 cases of meningitis, 42 urinary tract infections, 21 cases of malaria, 53 cases of tuberculosis, 27 cases of sepsis, 18 cases of severe anemia, 52 cases of unknown febrile illness, and 42 unknown causes of death in community.

MUAC categories were associated with protective associations similar to WHZ during follow-up (Table 2, **Supplemental Figure 1**). However, there was no reduction in the risk of LTEs associated with HAZ categories at these time points (**Supplemental Table 4, Supplemental Figure 2**). The HRs for specific LTEs are shown in Table 2.

To further investigate a potential time lag in the effect of nutritional recovery on the susceptibility to LTEs, the aHRs associated with continuous WHZ was compared between months 1, 3, and 6. At months 3 and 6, aHRs per unit of WHZ were lower than at month 1 (*P* < 0.003) (**Supplemental Table 5**). There was no evidence that the aHRs per unit of HAZ differed between months 1, 3, and 6 (*P* = 0.77) (Supplemental Table 5, Supplemental Figure 2).

WHZ at enrollment, but not age, sex, size at birth, index admission diagnosis, rural or urban site, and all other variables examined, was associated with WHZ category after 1, 3, 6, and 12 mo (**Supplemental Tables 6–9**). However, neither WHZ nor HAZ at enrollment were associated with subsequent LTEs during the following 6 or 12 mo (Figure 3A, Supplemental Table 4, and Supplemental Figure 2A), and WHZ at intermediate months did not alter the effects of WHZ measured at 1, 3, and 6 mo on subsequent LTEs (**Supplemental Table 10**).

### Association between nutritional status during follow-up and case fatality of LTEs

The overall case fatality ratio for LTEs was 257/823 (31%; 95% CI: 28%, 34%) and did not significantly change over time (*P* = 0.22). Children with a WHZ <−3 consistently had higher case fatality ratios for LTEs, which also did not change with time (*P* = 0.86; Supplemental Table 3).

## DISCUSSION

After treatment for complicated SAM in the absence of HIV infection, higher WHZ or MUAC during follow-up was associated with a strikingly lower risk of subsequent LTEs. Not surprisingly, the major factor at enrollment that was associated with WHZ at follow-up was WHZ at enrollment, but importantly, in this group initially selected by MUAC, WHZ at enrollment was not associated with the risk of LTEs, indicating that a child's capacity to respond to treatment is key to risk reduction.

Compared with being severely wasted, there were relatively small differences in risk between becoming moderately wasted and becoming nonwasted, suggesting that some WHZ recovery is a highly worthwhile target. The association between anthropometric measures during recovery and serious illness could be operating in either direction: it is plausible that illness episodes limited nutritional recovery through anorexia and diversion and losses of nutrients (17, 18). On the other hand, those remaining wasted may have had an increased risk of infection compared with those who partially or fully recovered.

The 15 LTEs/100 CYOs among children not classified as wasted by WHZ 6 mo after hospitalization that we observed is a far higher number than the incidence of hospitalization reported in other contexts in Africa. Among Tanzanian infants, the incidence of hospital admission within the first year of life was 4.32/100 CYOs in rural and 3.72/100 CYOs in urban (19) settings. In Bamako, Mali, 5.3 children/100 CYOs were hospitalized during the first year of life and 0.64/100 CYOs between ages 1 and 5 y (20). The incidence we observed is also higher than among Zambian children with HIV infection being treated with antiretroviral therapy and cotrimoxazole prophylaxis (8 hospital admissions/100 CYOs during 12 mo of follow-up) (21, 22). Thus, children with complicated SAM remain highly susceptible to life-threatening illness despite nutritional recovery. This could be due to ongoing immunologic dysfunction, untreated or partially treated infections such as tuberculosis or bacterial infections with reduced sensitivity to antimicrobials, other illnesses, poverty, or other unfavorable household- and community-level exposures.

The increased reduction in risk of LTEs per unit of WHZ from month 1 to month 6 (Supplemental Table 5) shows a time lag in recovery from susceptibility after treatment. Very few studies have investigated the dynamics of illness episodes or physiologic and immunologic recovery after treatment of malnutrition. In Brazil, during treatment for SAM following WHO guidelines, phagocytic function improved, but lymphocyte counts and the production of free radicals remained lower than in well-nourished controls (23). Malnourished children typically have thinning of the intestinal mucosa, shorter villi, loss of goblet cells, infiltration of lymphocytes, and both intestinal and systemic inflammation and are vulnerable to bacterial translocation (24–26). However, Chilean children recovering from SAM retained histologic evidence of damaged intestinal mucosa, although there was improvement shown by electron microscopy of the intestinal brush border (27). There is also evidence that the intestinal microbiome is important in the susceptibility to, and recovery from, malnutrition (28). Components of the microbiome are key to nutrient processing and also influence programming of the immune system. In Uganda, circulating leptin, a marker of white adipose tissue that is associated with energy production during infection and long-term mortality, did not change significantly after hospital discharge (29). These studies, although limited, suggest longer-term effects of malnutrition itself, as well as nutrition and care provision at home or living conditions.

In our study, after 6 mo of follow-up, only 56% children were not classified as wasted by a WHZ <−2. This was similar to the overall proportion of nutritionally “cured” children among those hospitalized with SAM in Malawi (including both complicated and uncomplicated cases by current definitions); however, this proportion ranged between 37% among HIV-infected children and 71% among non–HIV-infected children (30). In contrast, children with uncomplicated SAM treated in the community before the onset of serious illness typically have much higher recovery rates, with an estimated pooled proportion recovered of 88% (31). We were not able to ascertain adherence to ready-to-use therapeutic foods after hospital discharge; low adherence could partly explain the low recovery in this study.

Notably, children who missed some follow-up visits had elevated mortality risks, suggesting that data were not “missing at random” and were important to include in mortality estimates. This is likely to relate to illness, social or family problems, or reduced access to, or engagement with, medical services.

Our results raise the hypotheses that mortality after complicated SAM could be reduced by specific interventions to detect, investigate, and manage children with a suboptimal anthropometric response and that a broader set of vulnerabilities are indicated by an episode of complicated SAM that will need to be tackled to reduce the overall mortality associated with this condition.

### Strengths and limitations

The context of a randomized controlled trial is both a strength and a limitation. The major strengths of this study are rigorous follow-up, very low drop out (5%), well-documented episodes of LTEs, monthly anthropometric measurements, and a large sample size.

However, following up children in a clinical trial could have led to either over- or underreporting of LTEs. The free clinical services offered to participants could have led to more children being examined by the study clinicians and hospitalized. On the other hand, the availability of early treatment for mild episodes may have prevented more serious disease episodes. Defining common life-threatening infections with the use of the WHO clinical criteria, which are sensitive rather than specific, could have included noncases or excluded some cases (32). Use of data assigned by standardized verbal autopsy for community deaths, and from medical records when LTEs or deaths occurred in nonstudy hospitals. LTEs occurring in nonstudy hospitals could have affected the final diagnosis assigned, although not the fact that an LTE occurred.

Another possible limitation of the study is that we did not know the children's nutritional and immunologic status before the index hospitalization or changes in household exposures during recovery, which would have influenced the child's susceptibility, nutritional recovery, and long-term survival (33). Our results could also have been affected by survival bias. Excluding children with HIV means that we cannot generalize study results to children with SAM and HIV, a common feature in Africa, with known elevated mortality risk (6).

Future research is needed to better characterize anthropometric recovery in relation to immunologic and physiologic mechanisms of extended susceptibility to life-threatening disease, and in relation to longer term health outcomes (34). The role of nonnutritional factors needs further exploration, including undiagnosed infection, nosocomial acquisition of resistant pathogens, underlying chronic noninfectious conditions, and the social environment.

### Conclusions

Better nutritional status during recovery was associated with a rapid and substantial reduction in risk of LTEs. However, the reduction in susceptibility lagged behind anthropometric recovery and children who had “recovered” still had much higher risks than the general community. Research should ultimately guide evidence-based identification and intervention for children who respond poorly to treatment, because they are the most likely to become seriously ill or die. Monitoring disease episodes, together with anthropometric changes, may provide a more comprehensive picture of the true efficacy of nutrition-specific and allied interventions.

## Supplementary Material

Supplemental data

## Notes

Supported by the Wellcome Trust grant WT083579MA, awarded to JAB. JAB and MMN are currently supported by the Bill & Melinda Gates Foundation as part of the CHAIN study of childhood acute illness and nutrition (CHAIN grant OPP1131320). The funders had no role in the design, conduct, analysis, or writing of this manuscript.

This article was published with the permission of the Director of the Kenya Medical Research Institute.

Supplemental Tables 1–10 and Supplemental Figures 1 and 2 are available from the “Supplementary data” link in the online posting of the article and from the same link in the online table of contents at https://academic.oup.com/ajcn/.

Abbreviations used: aHR, adjusted HR; CYO, child-year of observation; HAZ, height- or length-for-age *z* score; LTE, life-threatening event; MUAC, midupper arm circumference; SAM, severe acute malnutrition; WHZ, weight-for-height or -length *z* score.
