# Supplementary material for: Changes in susceptibility to life-threatening infections after treatment for complicated severe malnutrition in Kenya
Source: Am J Clin Nutr. 2018 Apr 9;107(4):626–34. doi: 10.1093/ajcn/nqy007 (PMC6134064; doi:10.1093/ajcn/nqy007)
Supplement: Supplemental data [file nqy007_supp.docx]

# **Supplemental Table 1**: Anthropometric categories at study enrolment, months 1, 3, 6 and 12.

| **Characteristics** | **Enrolment** | **Month 1** | **Month 3** | **Month 6** | **Month 12** |
| --- | --- | --- | --- | --- | --- |
| Participants in follow-up- N (%) | 1778 | 1654 | 1569 | 1510 | 1429 |
| WHZ ≥-2 | 197 (11) | 557 (34) | 764 (49) | 842 (56) | 930 (65) |
| WHZ -2 to -3 | 373 (21) | 408 (25) | 306 (20) | 261 (17) | 200 (14) |
| WHZ<-3 | 908 (51) | 551 (34) | 308 (20) | 204 (13) | 124 (8.6) |
| Oedema | 300 (17) | 17 (1.0) | 3 (0.2) | 3 (0.2) | 3 (0.2) |
| Missing WHZ data | 5 (0.3) | 138 (8.3) | 191 (12) | 203 (13) | 172 (12) |
|  | | | | |  |
| MUAC≥12.5cms | 67 (3.8) | 239 (15) | 606 (39) | 828 (55) | 1016 (71) |
| MUAC 12.5 to 11.5cms | 108 (6.1) | 479 (29) | 446 (29) | 330 (22) | 158 (11) |
| MUAC<11.5cms | 1603 (90) | 843 (51) | 373 (24) | 181 (12) | 72 (4.9) |
| Missing MUAC data | - | 93 (5.6) | 144 (9.2) | 171 (11) | 183 (13) |
|  |  |  |  |  |  |
| HAZ ≥-2 | 548 (31) | 384 (23) | 346 (22) | 348 (23) | 329 (23) |
| HAZ -2 to -3 | 442 (25) | 376 (23) | 367 (24) | 365 (24) | 359 (25) |
| HAZ<-3 | 784 (44) | 758 (46) | 665 (43) | 595 (40) | 534 (37) |
| Missing HAZ data | 4 (0.2) | 136 (8.2) | 191 (12) | 202 (13) | 207 (14) |
| WHZ- Weight-for-height z-score, MUAC- Mid upper arm circumference, HAZ-Height-for-age z-score, reported results are N (proportion). | | | | | |

**Supplemental Table 2:** Incidence rate of life-threatening events during one year follow-up

| **Time of follow-up** | **Child-time (years)** | **Incidence rate of any life-threatening episodes per child-year (95% CI)** | **Incidence rate of severe pneumonia episodes per child-year (95% CI)** | **Incidence rate of diarrhoea episodes per child-year (95% CI)** | **Incidence rate of other diagnosis episodes per child-year (95% CI)** |
| --- | --- | --- | --- | --- | --- |
| Overall  (one-year follow-up) | 1556.4 | 0.54 (0.51, 0.58) | 0.21 (0.19, 0.23) | 0.10 (0.09, 0.12) | 0.10 (0.08, 0.12) |
| Month one | 141.5 | 1.56 (1.36, 1.77) | 0.51 (0.40, 0.64) | 0.27 (0.20, 0.37) | 0.16 (0.11, 0.24) |
| Month two | 134.6 | 0.88 (0.74, 1.06) | 0.36 (0.27, 0.47) | 0.16 (0.10, 0.24) | 0.18 (0.12, 0.27) |
| Month three | 130.9 | 0.59 (0.47, 0.74) | 0.28 (0.20, 0.38) | 0.11 (0.07, 0.19) | 0.12 (0.07, 0.20) |
| Month four | 128.0 | 0.61 (0.49, 0.76) | 0.23 (0.16, 0.33) | 0.14 (0.09, 0.22) | 0.14 (0.09, 0.22) |
| Month five | 126.0 | 0.65 (0.52, 0.81) | 0.33 (0.24, 0.44) | 0.14 (0.09, 0.23) | 0.12 (0.07, 0.20) |
| Month six | 124.8 | 0.38 (0.28, 0.50) | 0.18 (0.12, 0.27) | 0.06 (0.03, 0.12) | 0.10 (0.05, 0.17) |
| Month seven | 123.5 | 0.36 (0.27, 0.49) | 0.13 (0.08, 0.21) | 0.07 (0.04, 0.14) | 0.05 (0.02, 0.10) |
| Month eight | 122.6 | 0.27 (0.19, 0.38) | 0.11 (0.06, 0.18) | 0.06 (0.03, 0.12) | 0.05 (0.02, 0.11) |
| Month nine | 121.4 | 0.36 (0.27, 0.49) | 0.12 (0.07, 0.19) | 0.09 (0.05, 0.16) | 0.12 (0.07, 0.21) |
| Month ten | 120.3 | 0.22 (0.15, 0.32) | 0.07 (0.03, 0.13) | 0.02 (0.01, 0.07) | 0.05 (0.02, 0.11) |
| Month eleven | 119.3 | 0.24 (0.17, 0.35) | 0.08 (0.04, 0.14) | 0.03 (0.02, 0.10) | 0.04 (0.02, 0.10) |
| Month twelve | 118.0 | 0.18 (0.12, 0.27) | 0.08 (0.05, 0.16) | 0.04 (0.02, 0.10) | 0.04 (0.02, 0.10) |
| Incidence rates represents number of events per child-years (95% confidence intervals). | | | | | |

**Supplemental Table 3:** Incidence and outcome of subsequent life threatening events by WHZ categories assessed at months 1, 3 and 6.

|  | **WHZ ≥-2** | **WHZ -2 to -3** | **WHZ <-3** | **Missing WHZ** |
| --- | --- | --- | --- | --- |
| **After month 1** | | | | |
| Life-threatening events-N | 104 | 104 | 210 | 30 |
| CYO | 269.3 | 195.9 | 251.6 | 54.0 |
| Incidence rate per CYO (95% CI) | 0.39 (0.32, 0.47) | 0.53 (0.44, 0.64) | 0.83 (0.73, 0.96) | 0.55 (0.39, 0.79) |
| Fatal LTEs | 22 | 16 | 58 | 18 |
| Case fatality ratio (95% CI) | 0.21 (0.14, 0.30) | 0.15 (0.09, 0.24) | 0.28 (0.22, 0.34) | 0.60 (0.41, 0.77) |
| **After month 3** | | | | |
| Life-threatening events-N | 99 | 52 | 135 | 36 |
| CYO | 376.8 | 148.2 | 139.6 | 84.5 |
| Incidence rate per CYO (95% CI) | 0.26 (0.22, 0.32) | 0.35 (0.27, 0.46) | 0.97 (0.82, 1.14) | 0.43 (0.31, 0.59) |
| Fatal LTEs | 14 | 11 | 35 | 13 |
| Case fatality ratio (95% CI) | 0.14 (0.08, 0.23) | 0.21 (0.11, 0.35) | 0.26 (0.19, 0.34) | 0.36 (0.21, 0.54) |
| **After month 6** | | | | |
| Life-threatening events-N | 64 | 36 | 69 | 29 |
| CYO | 415.2 | 126.1 | 96.1 | 89.9 |
| Incidence rate per CYO (95% CI) | 0.15 (0.12, 0.20) | 0.29 (0.21, 0.40) | 0.72 (0.57, 0.91) | 0.32 (0.22, 0.46) |
| Fatal LTEs | 9 | 10 | 17 | 11 |
| Case fatality ratio (95% CI) | 0.14 (0.07, 0.25) | 0.28 (0.14, 0.45) | 0.25 (0.15, 0.36) | 0.38 (0.21. 0.58) |
| WHZ-Weight-for-height z-score, CYO-Child Year of observation, LTEs- Life-threatening events, Incidence rate represents events per child-years, Case fatality ratio represents proportion of children with LFEs who died. | | | | |

**Supplemental Table 4:** Hazard ratios for subsequent life-threatening events by height/length-for-age z-score categories assessed at months 1, 3 and 6.

| **HAZ** | **HAZ ≥-2 (95% CI)^1^** | **HAZ -2 to -3 (95% CI)^1^** | **Missing HAZ (95% CI)^1^** | **HAZ <-3** |
| --- | --- | --- | --- | --- |
| **Study enrolment** | | | | |
| All life-threatening episodes | 0.84 (0.68, 1.03) | 0.96 (0.78, 1.18) | None missing | 1.0 |
| Death | 0.76 (0.56, 1.04) | 0.73 (0.52,1.03) |  | 1.0 |
| Severe pneumonia | 0.72 (0.55, 0.96) | 0.93 (0.71, 1.23) |  | 1.0 |
| Severe diarrhea | 0.98 (0.66, 1.45) | 1.30 (0.89, 1.91) |  | 1.0 |
| Others^2^ | 0.97 (0.70, 1.37) | 0.96 (0.67, 1.36) |  | 1.0 |
| **At month 1** | | | | |
| All life-threatening episodes | 0.85 (0.65, 1.10) | 0.79 (0.61, 1.03) | 0.86 (0.56, 1.33) | 1.0 |
| Death | 0.70 (0.42, 1.16) | 0.79 (0.49, 1.29) | 2.20 (1.26, 3.83) | 1.0 |
| Severe pneumonia | 0.82 (0.58, 1.16) | 0.73 (0.51, 1.05) | 0.51 (0.25, 1.04) | 1.0 |
| Severe diarrhea | 0.89 (0.53, 1.47) | 0.90 (0.53, 1.52) | 0.59 (0.24, 1.48) | 1.0 |
| Others^2^ | 1.08 (0.70, 1.67) | 1.01 (0.64, 1.58) | 1.65 (0.93, 2.93) | 1.0 |
| **At month 3** | | | | |
| All life-threatening episodes | 0.77 (0.55, 1.07) | 0.82 (0.59, 1.13) | 0.82 (0.54, 1.23) | 1.0 |
| Death | 0.44 (0.22, 0.90) | 0.68 (0.37, 1.24) | 1.39 (0.74, 2.62) | 1.0 |
| Severe pneumonia | 0.84 (0.54, 1.30) | 0.86 (0.55, 1.35) | 0.60 (0.32, 1.11) | 1.0 |
| Severe diarrhea | 0.67 (0.37, 1.22) | 0.92 (0.53, 1.58) | 0.16 (0.04, 0.65) | 1.0 |
| Others^2^ | 0.81 (0.46, 1.42) | 0.70 (0.38, 1.27) | 0.99 (0.51, 1.91) | 1.0 |
| **At month 6** | | | | |
| All life- threatening episodes | 0.68 (0.44, 1.05) | 0.90 (0.60, 1.35) | 1.03 (0.62, 1.71) | 1.0 |
| Death | 0.77 (0.36, 1.63) | 0.46 (0.19, 1.11) | 1.61 (0.79, 3.30) | 1.0 |
| Severe pneumonia | 0.60 (0.30, 1.20) | 0.89 (0.50, 1.60) | 0.57 (0.27, 1.23) | 1.0 |
| Severe diarrhea | 0.54 (0.26, 1.13) | 0.73 (0.37, 1.48) | 0.46 (0.13, 1.59) | 1.0 |
| Others^2^ | 0.84 (0.41, 1.71) | 0.98 (0.49, 1.96) | 1.77 (0.87, 3.60) | 1.0 |
| The results are adjusted Hazard ratios (95% confidence intervals), HAZ-height/length-for-age z-score, ^1^adjusted for age, gender, recruitment site and randomization group, ^2^4 cases of bacteremia, 15 meningitis, 42 urinary tract infection, 21 Malaria, 53 tuberculosis, 27 sepsis, 18 severe anaemia, 52 unknown febrile illness and 42 unknown causes of death in community. | | | | |

**Supplemental Table 5:** Hazard ratios for subsequent life-threatening events per unit weight-for-height z-score, absolute MUAC and height-for-age z score assessed at months 1, 3 and 6 of follow-up.

|  | **WHZ (95% CI)** | **MUAC (95% CI)** | **HAZ (95% CI)** |
| --- | --- | --- | --- |
|  | **After month one** | |  |
| All life-threatening episodes | 0.79 (0.74, 0.86)^1^ | 0.77 (0.71, 0.85)^2^ | 0.93 (0.87, 0.99)^3^ |
| Death | 0.87 (0.75, 1.01) | 0.84 (0.70, 1.01) | 0.80 (0,71, 0.90) |
| Severe pneumonia | 0.78 (0.70, 0.86) | 0.77 (0.68, 0.88) | 0.92 (0.85, .01) |
| Severe diarrhoea | 0.73 (0.63, 0.85) | 0.87 (0.71, 1.06) | 0.98 (0.87, 1.12) |
| Others^4^ | 0.77 (0.67. 0.88) | 0.75 (0.64, 0.89) | 0.99 (0.89, 1.12) |
|  | **After month three** | |  |
| All life-threatening episodes | 0.68 (0.63, 0.74)^1^ | 0.67 (0.61, 0.74)^2^ | 0.90 (0.83, 0.98)^3^ |
| Death | 0.67 (0.55, 0.81) | 0.66 (0.53, 0.81) | 0.77 (0.65, 0.91) |
| Severe pneumonia | 0.65 (0.58, 0.74) | 0.66 (0.57, 0.75) | 0.92 (0.83, 1.04) |
| Severe diarrhoea | 0.69 (0.59, 0.82) | 0.70 (0.58, 0.85) | 0.94 (0.81, 1.09) |
| Others^4^ | 0.70 (0.60, 0.82) | 0.69 (0.58, 0.82) | 0.91 (0.79, 1.06) |
|  | **After month six** | |  |
| All life- threatening episodes | 0.64 (0.57, 0.72)^1^ | 0.66 (0.58, 0.75)^2^ | 0.94 (0.84, 1.04)^3^ |
| Death | 0.50 (0.40, 0.62) | 0.48 (0.38, 0.61) | 0.77 (0.61, 0.97) |
| Severe pneumonia | 0.57 (0.48, 0.68) | 0.63 (0.52, 0.77) | 0.96 (0.81, 1.14) |
| Severe diarrhoea | 0.68 (0.54, 0.86) | 0.69 (0.53, 0.90) | 0.91 (0.73, 1.13) |
| Others^4^ | 0.57 (0.47, 0.69) | 0.53 (0.43, 0.65) | 0.94 (0.77, 1.16) |
| Results are adjusted Hazard ratios (95% confidence intervals), WHZ-Weight-for-height z-score; MUAC- Mid upper arm circumference; HAZ-Height-for-age z score; Hazard ratios were adjusted for age, gender, recruitment site and randomization group, ^4^4 cases of bacteraemia, 15 meningitis, 42 urinary tract infections, 21 Malaria, 53 tuberculosis, 27 sepsis, 18 severe anaemia, 52 unknown febrile illness and 42 unknown causes of death in community; ^1^ I^2^=82% and P<0.003; ^2^ I^2^=64% and P=0.06; ^3^ I^2^=10% and P=0.77 | | | |

**Supplemental Table 6:** Associations between study enrolment characteristics and nutritional status at month one.

|  | WHZ ≥-2 compared to WHZ< -3 | | | WHZ -3 to -2 compared to WHZ <-3 | | |
| --- | --- | --- | --- | --- | --- | --- |
| Characteristic | **Adjusted RRR** | **95% CI** | **P-value** | **Adjusted RRR** | **95% CI** | **P-value** |
| Gender (female) | 1.08 | 0.76, 1.53 | 0.66 | 0.97 | 0.71, 1.33 | 0.86 |
| Age 24 to 59 months | 1.0 | Reference |  | 1.0 | Reference |  |
| Age 12 to 23 months | 1.20 | 0.52, 2.79 | 0.67 | 0.67 | 0.32, 1.42 | 0.30 |
| Age 6 to 11 months | 0.66 | 0.28, 1.52 | 0.33 | 0.38 | 0.18, 0.80 | 0.01 |
| Age 2 to 5 months | 0.46 | 0.19, 1.11 | 0.08 | 0.31 | 0.14, 0.69 | 0.004 |
| Height/length-for-age z score | 0.98 | 0.87, 1.11 | 0.75 | 1.14 | 1.02, 1.27 | 0.02 |
| Weight-for-length/height z score^1^ | 6.37 | 5.11, 7.93 | <0.001 | 2.87 | 2.38, 3.45 | <0.001 |
| Clinical signs of rickets | 0.59 | 0.35, 1.01 | 0.05 | 1.02 | 0.68, 1.55 | 0.91 |
| Index admission for severe pneumonia | 0.69 | 0.48, 1.02 | 0.05 | 0.83 | 0.60, 1.16 | 0.28 |
| Index admission for diarrhoea | 1.29 | 0.89, 1.86 | 0.18 | 1.34 | 0.96, 1.86 | 0.08 |
| Born premature/underweight^2^ | 0.84 | 0.56, 1.27 | 0.41 | 0.94 | 0.64, 1.36 | 0.73 |
| Randomised to co-trimoxazole prophylaxis | 1.21 | 0.86, 1.69 | 0.27 | 0.96 | 0.71, 1.30 | 0.79 |
| Enrolled at urban study site | 0.84 | 0.53, 1.32 | 0.46 | 1.07 | 0.69, 1.65 | 0.78 |
| Primary caretaker as mother | 0.99 | 0.45, 2.19 | 0.99 | 0.78 | 0.39, 1.55 | 0.48 |
| House wall material-mud/iron sheet | 0.95 | 0.65, 1.39 | 0.79 | 1.07 | 0.76, 1.50 | 0.70 |
| Water source, non-tap | 1.01 | 0.69, 1.49 | 0.94 | 0.81 | 0.56, 1.16 | 0.24 |
| Type of toilet-community shared/none | 0.64 | 0.37, 1.12 | 0.12 | 0.80 | 0.48, 1.34 | 0.40 |
| WHZ-Weight-for-height z-score, RRR-relative risk ratios, ^2^gestation age <37 weeks or birth weight <2500grams, ^1^significant at α=0.05, Relative risk ratios are computed using multinomial logistic regression with WHZ <-3 as the reference. | | | | | | |

**Supplemental Table 7:** Associations between study enrolment characteristics and nutritional status at month three.

|  | WHZ≥-2 compared to WHZ< -3 | | | WHZ -3 to -2 compared to WHZ< -3 | | |
| --- | --- | --- | --- | --- | --- | --- |
| Characteristic | **Adjusted RRR** | **95% CI** | **P-value** | **Adjusted RRR** | **95% CI** | **P-value** |
| Gender (female) | 1.01 | 0.72, 1.40 | 0.99 | 1.05 | 0.73, 1.51 | 0.79 |
| Age 24 to 59 months | 1.0 | Reference |  | 1.0 | Reference |  |
| Age 12 to 23 months | 1.78 | 0.82, 3.87 | 0.14 | 1.04 | 0.48, 2.26 | 0.93 |
| Age 6 to 11 months | 0.88 | 0.40, 1.90 | 0.74 | 0.65 | 0.30, 1.40 | 0.27 |
| Age 2 to 5 months | 1.02 | 0.45, 2.35 | 0.96 | 0.57 | 0.24, 1.35 | 0.20 |
| Height-for-age z score | 1.07 | 0.95, 1.20 | 0.24 | 1.12 | 0.99, 1.26 | 0.08 |
| Weight-for-length/height z score^1^ | 2.76 | 2.32, 3.27 | <0.001 | 1.53 | 1.29, 1.83 | <0.001 |
| Clinical signs of rickets | 0.53 | 0.34, 0.83 | 0.006 | 1.04 | 0.64, 1.57 | 0.98 |
| Index admission for severe pneumonia | 0.74 | 0.52, 1.05 | 0.09 | 0.82 | 0.57, 1.20 | 0.31 |
| Index admission for diarrhoea | 2.01 | 1.41, 2.85 | <0.001 | 1.19 | 0.82, 1.72 | 0.36 |
| Born premature/underweight^2^ | 1.15 | 0.77, 1.72 | 0.48 | 1.07 | 0.70, 1.65 | 0.75 |
| Randomised to co-trimoxazole prophylaxis | 1.22 | 0.89, 1.69 | 0.22 | 1.17 | 0.83, 1.64 | 0.38 |
| Primary caretaker as mother | 0.34 | 0.15, 0.77 | 0.01 | 0.76 | 0.31, 1.91 | 0.57 |
| Enrolled at urban study site | 0.62 | 0.38, 1.02 | 0.06 | 0.56 | 0.33, 0.95 | 0.03 |
| House wall material-mud/iron sheet | 1.27 | 0.88, 1.83 | 0.20 | 1.53 | 1.04, 2.25 | 0.03 |
| Water source, non-tap | 0.98 | 0.68, 1.43 | 0.93 | 1.01 | 0.67, 1.50 | 0.98 |
| Type of toilet-community shared/none | 0.80 | 0.46, 1.38 | 0.42 | 0.67 | 0.38, 1.16 | 0.15 |
| WHZ-Weight-for-height z-score, RRR-relative risk ratios, ^2^gestation age <37 weeks or birth weight <2500grams, ^1^significant at α=0.05, Relative risk ratios are computed using multinomial logistic regression with WHZ <-3 as the reference. | | | | | | |

**Supplemental Table 8:** Associations between characteristics at study enrolment and nutritional status at month six

|  | WHZ≥-2 compared to WHZ< -3 | | | WHZ -3 to -2 compared to WHZ< -3 | | |
| --- | --- | --- | --- | --- | --- | --- |
| Characteristic | **Adjusted RRR** | **95% CI** | **P-value** | **Adjusted RRR** | **95% CI** | **P-value** |
| Gender (female) | 1.07 | 0.73, 1.55 | 0.74 | 1.29 | 0.86, 1.94 | 0.23 |
| Age 24 to 59 months | 1.0 | Reference |  | 1.0 | Reference |  |
| Age 12 to 23 months | 1.45 | 0.63, 3.30 | 0.38 | 1.07 | 0.44, 2.61 | 0.88 |
| Age 6 to 11 months | 0.89 | 0.39, 2.04 | 0.79 | 0.94 | 0.39, 2.29 | 0.90 |
| Age 2 to 5 months | 0.56 | 0.23, 1.33 | 0.19 | 0.67 | 0.26, 1.73 | 0.41 |
| Height-for-age z score | 1.14 | 1.01, 1.29 | 0.05 | 1.08 | 0.94, 1.25 | 0.27 |
| Weight-for-length/height z score^1^ | 2.33 | 1.95, 2.79 | <0.001 | 1.30 | 1.07, 1.56 | 0.007 |
| Clinical signs of rickets | 0.71 | 0.44, 1.16 | 0.17 | 0.92 | 0.55, 1.55 | 0.76 |
| Index admission for severe pneumonia | 0.69 | 0.47, 1.01 | 0.06 | 0.72 | 0.47, 1.09 | 0.12 |
| Index admission for diarrhoea | 1.91 | 1.31, 2.78 | 0.001 | 1.50 | 0.98, 2.28 | 0.06 |
| Born premature/underweight^2^ | 1.33 | 0.86, 2.06 | 0.21 | 0.94 | 0.57, 1.55 | 0.82 |
| Randomised to co-trimoxazole prophylaxis | 1.13 | 0.79, 1.61 | 0.51 | 1.24 | 0.84, 1.83 | 0.28 |
| Admitted to urban hospitals | 0.81 | 0.48, 1.37 | 0.43 | 0.93 | 0.52, 1.66 | 0.80 |
| Primary caretaker as mother | 0.55 | 0.23, 1.31 | 0.18 | 0.67 | 0.26, 1.71 | 0.40 |
| House wall material-mud/iron sheet | 1.65 | 1.09, 2.48 | 0.02 | 1.45 | 0.93, 2.28 | 0.10 |
| Water source, non-tap | 0.71 | 0.47, 1.05 | 0.09 | 0.77 | 0.49, 1.20 | 0.24 |
| Type of toilet-community shared/none | 0.71 | 0.39, 1.29 | 0.26 | 0.90 | 0.47, 1.73 | 0.75 |
| WHZ-Weight-for-height z-score, RRR-relative risk ratios, ^2^gestation age <37 weeks or birth weight <2500grams, ^1^significant at α=0.05, Relative risk ratios are computed using multinomial logistic regression with WHZ <-3 as the reference. | | | | | | |

**Supplemental Table 9:** Associations between characteristics at study enrolment and nutritional status at month twelve.

|  | WHZ≥-2 compared to WHZ< -3 | | | WHZ -3 to -2 compared to WHZ< -3 | | |
| --- | --- | --- | --- | --- | --- | --- |
| Characteristic | **Adjusted RRR** | **95% CI** | **P-value** | **Adjusted RRR** | **95% CI** | **P-value** |
| Gender (female) | 1.40 | 0.87, 2.25 | 0.16 | 2.09 | 1.22, 3.55 | 0.007 |
| Age 24 to 59 months | 1.0 | Reference |  | 1.0 | Reference |  |
| Age 12 to 23 months | 1.79 | 0.66, 4.84 | 0.25 | 1.24 | 0.42, 3.70 | 0.70 |
| Age 6 to 11 months | 1.06 | 0.40, 2.80 | 0.91 | 0.68 | 0.23, 2.02 | 0.49 |
| Age 2 to 5 months | 0.71 | 0.24, 2.01 | 0.50 | 0.84 | 0.26, 2.70 | 0.77 |
| Height-for-age z score | 1.07 | 0.92, 1.25 | 0.38 | 1.06 | 0.89, 1.27 | 0.49 |
| Weight-for-length/height z score^1^ | 2.52 | 2.01, 3.15 | <0.001 | 1.47 | 1.15, 1.88 | 0.002 |
| Clinical signs of rickets | 0.79 | 0.44, 1.40 | 0.42 | 0.72 | 0.37, 1.41 | 0.34 |
| Index admission for severe pneumonia | 0.67 | 0.42, 1.08 | 0.10 | 0.87 | 0.51, 1.49 | 0.61 |
| Index admission for diarrhoea | 1.97 | 1.23, 3.16 | 0.005 | 1.27 | 0.75, 2.16 | 0.38 |
| Born premature/underweight^2^ | 0.85 | 0.51, 1.44 | 0.55 | 0.80 | 0.44, 1.45 | 0.46 |
| Randomised to co-trimoxazole prophylaxis | 1.09 | 0.70, 1.69 | 0.71 | 1.18 | 0.72, 1.95 | 0.51 |
| Admitted to urban hospitals | 1.31 | 0.70, 2.45 | 0.40 | 1.12 | 0.55, 2.29 | 0.76 |
| Primary caretaker as mother | 0.48 | 0.15, 1.52 | 0.21 | 0.71 | 0.19, 2.64 | 0.61 |
| House wall material-mud/iron sheet | 1.19 | 0.71, 1.97 | 0.51 | 1.11 | 0.62, 1.97 | 0.73 |
| Water source, non-tap | 0.89 | 0.54, 1.48 | 0.65 | 0.71 | 0.39, 1.27 | 0.25 |
| Type of toilet-community shared/none | 0.77 | 0.38, 1.58 | 0.48 | 0.97 | 0.43, 2.22 | 0.95 |
| WHZ-Weight-for-height z-score, RRR-relative risk ratios, ^2^gestation age <37 weeks or birth weight <2500grams, ^1^significant at α=0.05, Relative risk ratios are computed using multinomial logistic regression with WHZ <-3 as the reference. | | | | | | |

**Supplemental Table 10:** Hazard ratios for subsequent life-threatening events during the following six months by weight-for-height z-score assessed at study enrolment, months 1, 3 and 6.

|  | **WHZ ≥-2 (95% CI)^1^** | **WHZ -2 to -3 (95% CI)^1^** | **Missing WHZ** | **WHZ <-3 or Oedema** |
| --- | --- | --- | --- | --- |
|  | **At study enrolement^3^** | | | |
| All Life-threatening episodes | 0.95 (0.74, 1.22) | 0.89 (0.73, 1.09) |  | 1.0 |
| Death | 0.71 (0.46, 1.11) | 0.80 (0.55, 1.17) |  | 1.0 |
| Severe pneumonia | 0.92 (0.65, 1.30) | 1.09 (0.84, 1.41) |  | 1.0 |
| Severe diarrhoea | 0.68 (0.42, 1.13) | 0.60 (0.39, 0.91) |  | 1.0 |
| Others^2^ | 0.94 (0.60, 1.48) | 0.88 (0.62, 1.24) |  | 1.0 |
|  | **WHZ ≥-2 (95% CI)^1^** | **WHZ -2 to -3 (95% CI)^1^** | **Missing WHZ** | **WHZ <-3** |
|  | After month one | | | |
| All Life-threatening episodes | 0.45 (0.35, 0.59) | 0.64 (0.50, 0.82) | 0.62 (0.42, 0.93) | 1.0 |
| Death | 0.40 (0.23, 0.70) | 0.41 (0.23, 0.72) | 1.49 (0.85, 2.60) | 1.0 |
| Severe pneumonia | 0.45 (0.31, 0.65) | 0.59 (0.42, 0.83) | 0.34 (0.17, 0.67) | 1.0 |
| Severe diarrhoea | 0.28 (0.15, 0.53) | 0.76 (0.48, 1.22) | 0.38 (0.15, 0.98) | 1.0 |
| Others^2^ | 0.32 (0.20, 0.51) | 0.43 (0.27, 0.67) | 0.87 (0.49, 1.53) | 1.0 |
|  | After month three | | | |
| All Life-threatening episodes | 0.29 (0.21, 0.40) | 0.38 (0.27, 0.52) | 0.48 (0.31, 0.72) | 1.0 |
| Death | 0.13 (0.06, 0.29) | 0.32 (0.16, 0.64) | 0.52 (0.23, 1.15) | 1.0 |
| Severe pneumonia | 0.37 (0.23, 0.57) | 0.42 (0.26, 0.66) | 0.43 (0.23, 0.83) | 1.0 |
| Severe diarrhoea | 0.32 (0.17, 0.60) | 0.50 (0.28, 0.91) | 0.64 (0.33, 1.24) | 1.0 |
| Others^2^ | 0.32 (0.18, 0.57) | 0.32 (0.17, 0.60) | 0.54 (0.27, 1.11) | 1.0 |
|  | After month six | | | |
| All Life-threatening episodes | 0.30 (0.19, 0.46) | 0.50 (0.32, 0.77) | 0.76 (0.47, 1.27) | 1.0 |
| Death | 0.17 (0.06, 0.47) | 0.61 (0.26, 1.41) | 1.29 (0.52, 3.23) | 1.0 |
| Severe pneumonia | 0.28 (0.14, 0.55) | 0.43 (0.23, 0.83) | 0.47 (0.19, 1.12) | 1.0 |
| Severe diarrhoea | 0.49 (0.21, 1.12) | 0.50 (0.22, 1.16) | 0.65 (0.21, 2.01) | 1.0 |
| Others^2^ | 0.16 (0.07, 0.35) | 0.37 (0.17, 0.80) | 0.80 (0.36, 1.78) | 1.0 |
| Results are adjusted Hazard ratios (95% confidence intervals), WHZ-Weight-for-height z-score, ^3^only 5(0.3%) were missing WHZ at enrolment, ^1^adjusted for age, gender, recruitment site, randomization group and previous months’ nutrition status, ^2^4 cases of bacteraemia, 15 meningitis, 42 urinary tract infections, 21 Malaria, 53 tuberculosis, 27 sepsis, 18 severe anaemia, 52 unknown febrile illness and 42 unknown causes of death in community. | | | | |

| **A**  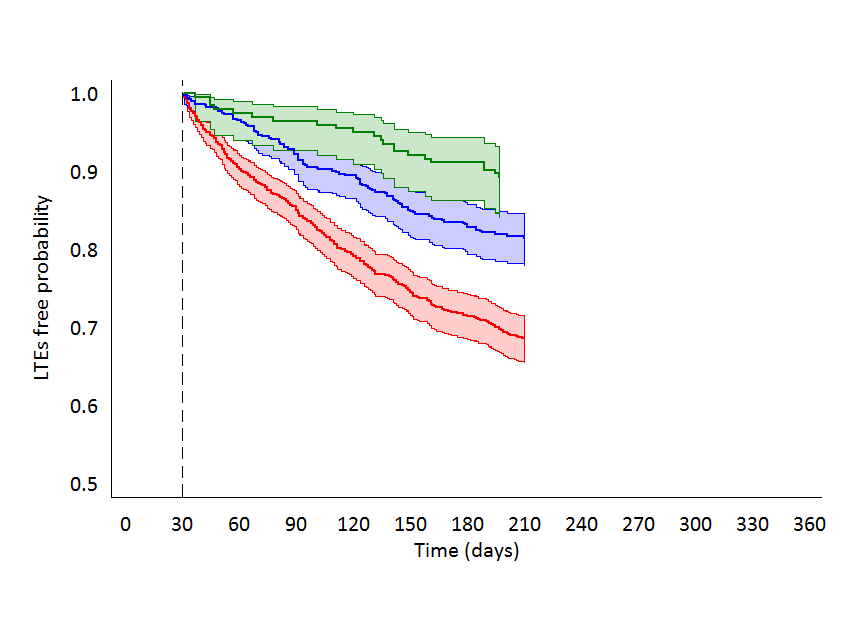 |
| --- |
| **B**  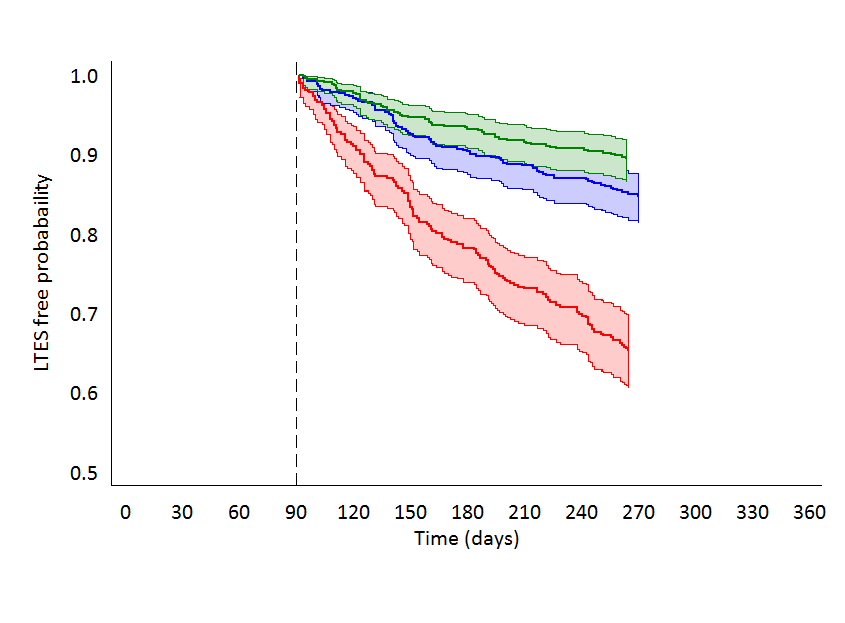 |
| **C**  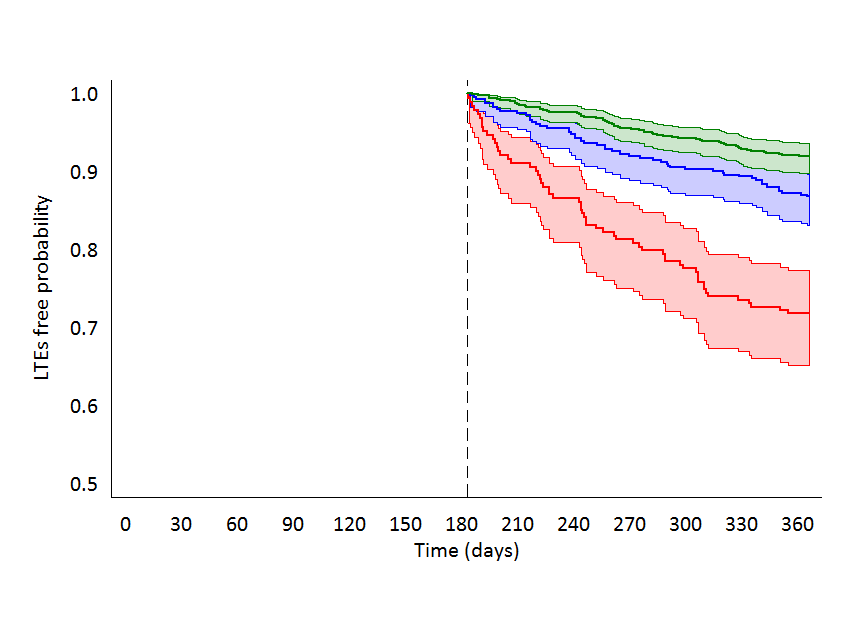 |
| (MUAC <11.5cm (Red), MUAC 12.5, 11.5cm (Blue) and MUAC ≥12.5cm (Green))  **Supplemental Figure 1:** Kaplan-Meier graphs for subsequent life-threatening events during the following six months by Mid-Upper Arm Circumference (MUAC) (95% CI) after:  **A**-month 1, **B**-month 3, and **C**-month 6. |

| A  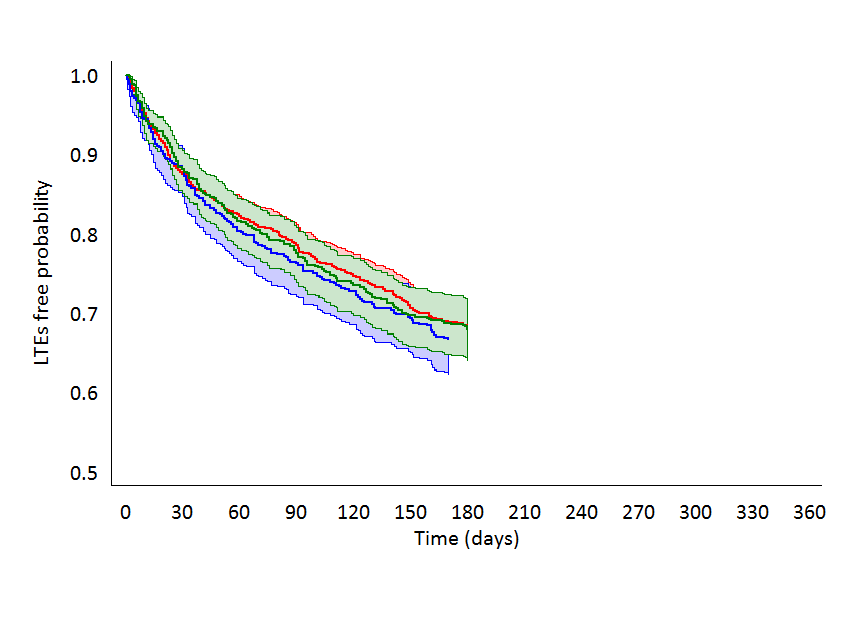 | **B**  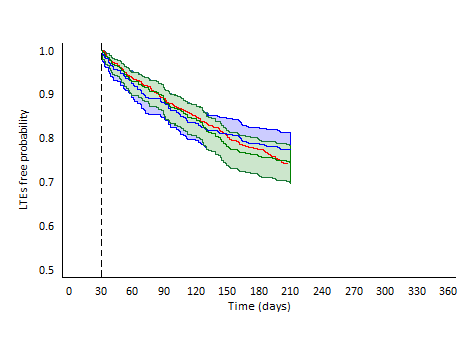 |
| --- | --- |
| **C**  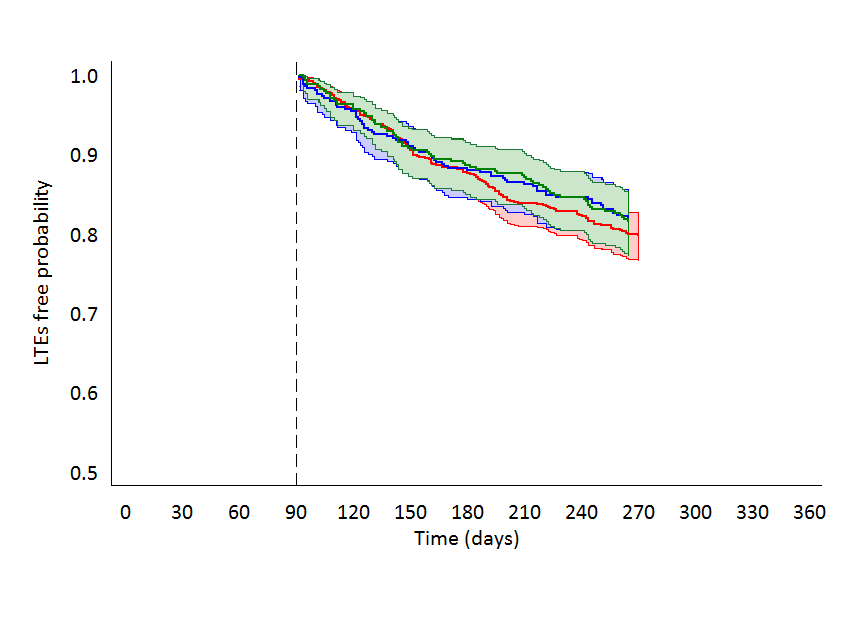 | **D**  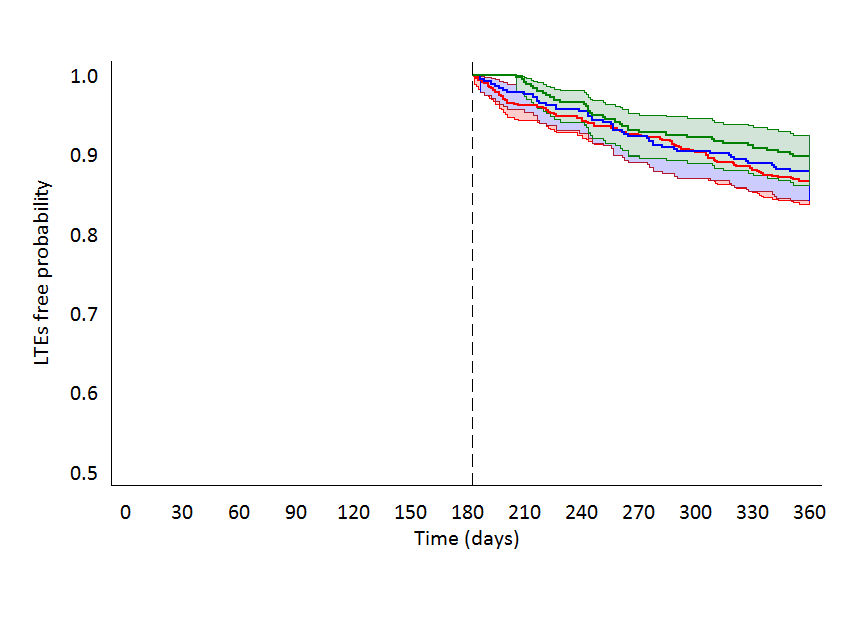 |
| (HAZ <-3 (Red), HAZ -2, -3 (Blue) and HAZ ≥-2 (Green)), HAZ-Haight/length-for-age z-score.  **Supplemental Figure 2:** Kaplan-Meier graphs for subsequent life-threatening events during the following six months by height-for-age z-scores (95% CI) at:  **A**-study enrolment, **B**-after month 1, **C**-after month 3 and **D**-after month 6. | |
